# A Novel HAGE/WT1-ImmunoBody^®^ Vaccine Combination Enhances Anti-Tumour Responses When Compared to Either Vaccine Alone

**DOI:** 10.3389/fonc.2021.636977

**Published:** 2021-06-28

**Authors:** Rukaia Almshayakhchi, Divya Nagarajan, Jayakumar Vadakekolathu, Barbara-Ann Guinn, Stephen Reeder, Victoria Brentville, Rachael Metheringham, A. Graham Pockley, Lindy Durrant, Stephanie McArdle

**Affiliations:** ^1^ John van Geest Cancer Research Centre, School of Science and Technology, Nottingham Trent University, Nottingham, United Kingdom; ^2^ Centre for Health, Ageing and Understanding Disease, School of Science and Technology, Nottingham Trent University, Nottingham, United Kingdom; ^3^ Department of Biomedical Sciences, University of Hull, Hull, United Kingdom; ^4^ Scancell Ltd, Biodiscovery Institute, University of Nottingham, Nottingham, United Kingdom

**Keywords:** WT1, ImmunoBody^®^, immunotherapy, HAGE, cancer vaccine combination, HHDII/DR1 mice

## Abstract

Many cancers, including myeloid leukaemia express the cancer testis antigen (CTA) DDX43 (HAGE) and/or the oncogene Wilms’ tumour (WT1). Here we demonstrate that HAGE/WT1-ImmunoBody^®^ vaccines derived T-cells can kill *ex-vivo* human CML cell lines expressing these antigens and significantly delay B16/HHDII^+^/DR1^+^/HAGE^+^/WT1^+^ tumour growth in the HHDII/DR1 mice and prolonged mouse survival in the prophylactic setting in comparison to non-immunised control mice. We show that immunisation of HHDII/DR1 mice with HAGE- and WT1-ImmunoBody^®^ DNA vaccines in a prime-boost regime in two different flanks induce significant IFN-γ release by splenocytes from treated mice, and a significant level of cytotoxicity against tumour targets expressing HAGE/WT1 *in vitro*. More importantly, the combined HAGE/WT1 ImmunoBody^®^ vaccine significantly delayed tumour growth in the B16/HHDII^+^/DR1^+^/HAGE^+^/WT1^+^ tumour model and prolonged mouse survival in the prophylactic setting in comparison to non-immunised control mice. Overall, this work demonstrates that combining both HAGE- and WT1-ImmunoBody^®^ into a single vaccine is better than either vaccine alone. This combination vaccine could be given to patients whose cancer expresses HAGE and WT1 in parallel with existing therapies in order to decrease the chance of disease progression and relapse.

## Introduction

The role of a cancer vaccine is to stimulate cellular immune responses that effectively lead to anti-tumour cytotoxic T-lymphocyte (CTL) responses. We have previously shown that DNA vaccines in which antigenic epitopes are encoded within an antibody framework can lead to T cell responses and delayed tumour growth (1). ImmunoBody(R) vaccines have entered Phase I clinical trials (2) and demonstrated safety as well as immune responses in 23/25 evaluable melanoma patients. Previous studies have incorporated epitopes from a single antigen to stimulate anti-tumour responses (3) but few have used epitope combinations that could widen the applicability of vaccines to stimulate both CD4+ and CD8+ T cell responses and recognise multiple tumour cell populations (2).

The oncogene Wilm’s Tumour (WT1) has been found to play a role in normal kidney, genital tract and eye development and be mutated in some Wilms’ tumours. In addition it has been found to be elevated in uterine leiomyosarcoma and carcinosarcomas (4) as well as epithelial ovarian cancer (5). However it is frequently used as a marker of minimal residual disease in chronic myeloid and acute leukaemia (6) having been found to be overexpressed in malignant but not healthy haematopoietic cells. WT1 is one of a number of leukaemia associated antigens that have been considered as promising targets for immunotherapy because of their ability to elicit specific immune responses against antigen-bearing cancerous cells while sparing normal tissues. A National Cancer Institute pilot project by Cheever et al. (7), developed a priority-ranked list of cancer vaccine target antigens based on predefined and weighted criteria for the ‘ideal’ antigen. Although no single antigen met all of the top subcriteria such as therapeutic function, immunogenicity and having already been in clinical trials, WT1 was identified as the top ranked antigen for these criteria and WT1 is overexpressed in most *de novo* acute myeloid leukaemia (AML) cases (8) and in chronic myeloid leukaemia (CML) (9). More specifically WT1 was shown to be expressed in 50–100% cases of blast crisis but not in chronic or accelerated phase cases (10).

HAGE is a cancer-testis antigen (CTA), a member of a family of HAGE was found to be expressed in (12/16) 75% of carcinomas (11) and in 57% of CML patient samples at diagnosis (12). Expression of HAGE, like many CTAs (13, 14), is limited in healthy tissues except immunologically protected sites such as the testis and placenta, making it an excellent target for immunotherapy due to its low associated risk of effective treatment causing damage to healthy tissues.

Leukaemia, and CML in particular, have gained a special attention in the field of the immunotherapy due to the fact that the circulating tumour cells are readily accessible to immune attack. Furthermore, the disease has a well-defined carcinogenesis pathway that allows for the development of immunotherapies targeting particular and well characterised targets. CML is a clonal myeloproliferative disorder resulting from malignant transformation of the primitive haematopoietic stem cell (HSC). The disease is characterised by the formation of a fusion gene, called *BCR/ABL1* which encodes a chimeric protein that has a constitutive oncogenic tyrosine kinase (TK) activity. Targeting this enzyme using tyrosine kinase inhibitors (TKIs) such as imatinib (15), which is often the first line ‘gold standard’ therapeutic approach, has significantly improved the clinical outcome for patients with CML. Despite this, 35-40% of patients with CML on TKIs develop resistance (16), often due to the clonal outgrowth of CML cells harbouring BCR-ABL point mutations (recently reviewed in (17). These findings demonstrate that most TKIs are not curative, but merely put CML stem cells into a state of autophagy (18). However, the pathognomonic molecular characteristics of CML and the particular nature of cancer-host immune cells interaction in CML, as well as the advantageous immunomodulatory effects of the imatinib therapy (19), all offer a favourable setting in which immunotherapeutic strategies could be added with synergistic effect. Indeed, active immunotherapeutic strategies that enhance T cell responses against specific antigens in patients on imatinib therapy could increase the number of patients experiencing relapse-free survival following cessation of imatinib.

Using data from CML single cell (SC) gene expression downloaded from GEO database (Acc. No. GSE76312 (20),) we were able to demonstrate that both HAGE and WT1 genes were expressed in CML cancer stem cells but not in HSC cells highlighting the specific tumour cell expression of these antigens (**Supplementary Figure 1**). Thus it made sense to assess the capacity of two sequences, one derived from DDX43 (HAGE) and the other from WT1, to generate effective anti-tumour immune responses in a humanised HHDRII/DR1 mouse model and determine the ability the ImmunoBody^®^ DNA delivery system (1) to induce tumour-specific cell death. Although ImmunoBody^®^ DNA can be administered *via* intradermal, intravenous, intramuscular, subcutaneous or intraperitoneal routes (21), the standard method is the intradermal route using a gene gun device in mice, and i.m. injection combined with electroporation in humans (2). The overall goal of this study was to evaluate the capacity of a combined HAGE/WT1 sequence-based vaccine to eliminate/prevent the growth of HAGE/WT1 expressing tumours. We hypothesised that combining both HAGE and WT1 vaccines would be more effective against cancerous cells expressing both antigens through the reduced chance of escape variants, and also that such a vaccine could be used against a wider range of cancers that express either, or both of these antigens.

This study has therefore explored and compared the following aspects; the immunogenicity of HAGE and WT1 derived ImmunoBody^®^ vaccines individually and in combination in HHDII/DR1 mice, the capacity of the vaccines given individually and in combination to induce activated cytotoxic T cells that can specifically recognise and kill HAGE/WT1-expressing targets *in vitro*, and finally, the *in vivo* capability of the combined vaccine to induce tumour rejection using HAGE^+^/WT1^+^ expressing humanised B16 cells as a “Proof-of-Concept” in tumour challenge experiments.

## Materials and Methods

### Animals

HHDII-DR1 double transgenic mice expressing human α1 and α2 chains of HLA-A*0201 chimeric with α3 chain of H-2Dd allele (HHDII), also expressing HLA-DRB*0101, and knocked out for the expression of murine MHC class I (H-2b) and II (I-Ab) were provided by Dr. Lone (CNRS, Orleans, France). Animal use and care was in accordance with EU Directive 2010/63/EU and UK Home Office Code of Practice for the housing and care of animals bred, supplied, or used for scientific purposes. The studies were undertaken with UK Home Office approval. Females aged between 6-8 weeks were used for this study. Three mice per group were used to assess the immunogenicity of the vaccine in at least two independent studies, whereas a minimum of 10 mice per group were used for tumour model experiments.

### Cell Lines and Growth Condition

The Transporter-Associated Proteins (TAP) deficient, HLA-A2+ lymphoblastoid suspension cell line, T2 (22), was a gift from Dr. J. Bartholomew (Paterson Institute, Manchester, UK). The CML derived cell line K562 was purchased from ATCC, the KCL-22 CML cell line was kindly provided by Anthony Nolan (UK, https://www.anthonynolan.org). T2 cells, KCL-22 and TCC-S cells were cultured in the Roswell Park Memorial Institute (RPMI) 1640 medium (SLS/Lonza, BE12-167F) containing 10% (v/v) foetal bovine serum (FBS, Life Technologies, 10270106) and 2mM L-Glutamine (SLS/Lonza, BE17-605E). K562 cells were cultured in Iscove’s modified Dulbecco’s medium (IMDM, SLS/Lonza, BE12-722F) containing 10% (v/v) FBS.

For the tumour implant model, the B16F1 (murine melanoma) cell line was knocked out for both murine endogenous MHC class I and II by Zinc Finger Nuclease (ZFN) technology and stably transfected with plasmids encoding both HHDII and HLA-DR1 molecules. Cells were grown in RPMI 1640 supplemented with 10% (v/v) FBS, 1% L-Glutamine, 300μg/mL Hygromycin (Insight Biotechnology, Middlesex, UK) and 500μg/mL Geneticin (Insight Biotechnology). The cells were further transfected with luciferase and HAGE constructs (23). In this study, the cells were referred to as _h_B16/HAGE^+^/Luc^+^, and maintained in RPMI-1640 medium supplemented with 10% (v/v) FBS, 1% L-Glutamine, 300μg/mL hygromycin and 550μg/mL zeocin (Thermo Fisher Scientific, U.K.) to retain luciferase gene expression, and 1μg/mL puromycin (Thermo Fisher Scientific) to retain HAGE gene expression. The HEK293T human embryonic kidney cell line (Thermo Fisher Scientific) was maintained in DMEM (SLS/Lonza, Yorkshire, U.K.) containing 10% (v/v) FBS and 2mM L-Glutamine - these cells were routinely used for viral gene transduction. All cells were maintained in a 37*°*C incubator with 5% (v/v) CO_2._

### Epitope Prediction

The WT1 protein sequence was screened for peptides that have binding affinity to HLA-A2 and HLA-DR1 molecules using a web-based algorithm (www.syfpeithi.de) (24) and peptides selected according to their binding score. A 15 amino acid long peptide (VRDLNALLPAVPSLG) derived from the WT1 protein was chosen. The HAGE 30mer sequence used in this study (QTGTGKTLCYLMPGFIHLVLQPSLKGQRNR) has previously been studied by our group (23), and the same class I and class II HAGE-derived peptides were used herein. All peptides were synthesised by GenScript Ltd (Piscataway, USA), with a minimum purity of 80%. All peptides were reconstituted in 100% (v/v) dimethyl sulfoxide (DMSO) to a stock concentration of 10mg/mL and stored at -80°C in small aliquots to minimise the number of freeze/thaw cycles.

### Peptide Binding Affinity Assay

A T2 peptide-binding assay was performed to evaluate the binding affinity of the HAGE and WT1 class I peptide sequences to the HLA-A2 molecules, as predicted by SYFPEITHI software. T2 cells are an HLA-A2 human lymphoblast suspension cell line that have been genetically modified to produce mutated non-functional TAPs that are necessary to transport MHC class I restricted peptides into the endoplasmic reticulum (ER). This defect results in a failure to present internal TAP-dependant peptides and instead MHC molecules leave the ER and the Golgi compartments empty, leading to a 70–80% reduction of HLA‐A2 expression on T2 cell surface. These empty molecules are not stable and are quickly recycled. However, the empty HLA-A2 molecules on the surface of cells can be stabilised by addition of exogenous peptides for which they have an affinity (25). T2 cells are therefore frequently used to validate the binding of exogenously administered peptides. Briefly, in a sterile 96-well rounded-bottom plate, T2 cells were plated at 0.5×10^6^ cells/100µL in serum-free RPMI medium supplemented with 3µg/mL human β2m (Sigma-Aldrich, Hertfordshire, U.K.) in the presence of varying concentrations of HAGE and WT1 class I peptides or with DMSO as negative controls and then incubated at 26°C overnight. T2 cells were then harvested, washed and stained with an APC-conjugated human HLA-A2 monoclonal antibody (BioLegend, 343308, Clone: BB7.2) for 30 minutes at 4°C. Cells were then washed and stained with propidium iodide (PI, Sigma-Aldrich) to exclude dead cells, and then immediately run to assess HLA-A2 expression using a Beckman Coulter Gallios™ flow cytometer and Kaluza™ data acquisition and analysis software.

### Brefeldin A Decay Assay (BFA)

A BFA decay assay was performed to evaluate the stability of the class I peptide-HLA-A2 complex on the cell surface. For this, T2 cells were seeded in a 96-well rounded-bottom plate at a concentration of 1×10^6^ cells/100 µL in a serum-free RPMI medium containing 3µg/mL human β2m. Cells were cultured with either the candidate peptides at a final concentration of 50µg (as determined by T2 binding assay) or with DMSO as a negative control and incubated at 26°C overnight. The next day, one batch of cells was washed and incubated with 10µg/mL BFA (BioLegend, 420601) for 1 hour at 37*°*C (Time 0) and thereafter every two hours until 8 hours. After each incubation, cells were transferred into 12x75 mm polycarbonate tubes, washed and stained with an APC-conjugated human HLA-A2 monoclonal antibody (BioLegend, 343308, Clone: BB7.2) for 30 minutes at 4°C and analysed using the flow cytometer. Dead cells were excluded by staining with LIVE/DEAD™ viability dye (Invitrogen) or PI (Sigma-Aldrich).

The stability of peptide-HLA-A2 complex was assessed by calculating the DC_50_ value, which is defined as the time required for a 50% reduction in the level of MFI recorded at time 0 (26), the longer the time for this to happen, the more stable the complex. The DC_50_ was calculated according to the formula: (MFI at a given time point/MFI at time 0) X100%. T2 cells incubated in the absence of peptide, but in the presence of the same concentrations of DMSO were used as negative controls.

### Target Cell Line Preparation: Gene Knock-In and Knock Down

The CML derived cell lines, K562, KCL-22 and TCC-S were genetically engineered to either over express HAGE and/or WT1 proteins or HAGE and/or WT1 expression was silenced. K562 cells constitutively express low levels of HLA and were electroporated with the pcDNA-3.1/HHDII gene using SF cell line 4D Nucleofection™ Xkit/Amaxa according to manufacturer’s instructions. Positively transfected cells were selected using 2000µg/mL of G418. K562/HHDII^+^ cells and KCL-22 cells were transduced with HAGE using a PLenti.Puro/HAGE plasmid. An empty plasmid control PLenti.Puro/empty vector, was used as a vector control. Cells were selected with 1µg/mL puromycine and 2000µg/mL G418 in a 24-well plate at 37°C in 5% (v/v) CO_2_. Gene silencing was performed using short hairpin RNA (shRNA) sequences targeting the human WT1 (MISSION shRNA/Sigma-Aldrich in TCC-S cells

(WT1.shRNA_1_,TRCN0000010466,CGGATGAACTTAGGAGCCACCTTCTCGAGAAGGTGGCTCCTAAGTTCATCTTTTTG and WT1.shRNA_2_, TRCN0000040064,

CCGGGCAGTGACAATTTATACCAAACTCGAGTTTGGTATAAATTGTCACTGCTTTTTG).

After have being transduced, cells were selected with 1µg/mL puromycin and assessed for either HAGE or WT1 expression using Western blot and RT-qPCR.

### Real-Time Quantitative Polymerase Chain Reaction (RT-qPCR)

RT-qPCR was used to assess the expression of the genes of interest in target cells. Briefly, total RNA was isolated from cell lines using a RNeasy Mini Kit (Qiagen, Lancashire, UK), following the manufacturer’s instructions. The quantity and purity of the produced RNA was evaluated using a NanoDrop™ 8000 Spectrophotometer (ThermoFisher Scientific). 2μg of RNA was then reverse transcribed into cDNA using Promega M-MLV Reverse Transcriptase. For each qPCR reaction, 1µL of cDNA (diluted 1:1 in ddH_2_O) was mixed with 6.75µL of SYBR™ Green Supermix (Bio-Rad, 172-5124), 0.5µL (5pmol) of the forward, 0.5µL (5pmol) of the reverse primer (Sigma-Aldrich) and 3.25µL of molecular grade water. Each sample was run in triplicate on a Rotor-Gene Q real-time PCR cycler (Qiagen). **Table 1** below summarises primer pair sequences and annealing temperatures used in the PCR reaction. It also shows the HAGE-codon optimised pair primers which were used to detect the transduced HAGE gene in the modified cells. Expression levels of each genes were calculated using comparative CT method (27).

**Table 1:** Primers and annealing temperatures used in RT-qPCR assay.

Primers	Sequence	Annealing Temp
**WT1 forward**	GACTCATACAGGTGAAAAGC	58°C
**WT1 reverse**	GAGTTTGGTCATGTTTCTCTG	58°C
**DDX43 forward**	CAACACCTATTCAGTCACAG	58°C
**DDX43 reverse**	GACCAGATGAATAAATCCAGG	58°C
**GUSB forward**	ACTGAACAGTCACCGAC	58°C
**GUSB reverse**	AAACATTGTGACTTGGCTAC	58°C
**HAGE forward (codon optimised)**	CCACATGCACTTTCGACGAT	58°C
**HAGE reverse (codon optimised)**	ATTCCTGGTCGGTTCCTCTG	58°C

### Protein Assay and Western Blot Analysis

Immunoblotting was performed to detect HAGE and/or WT1 expression in target cells. Briefly, CML cells were washed twice with cold DPBS, lysed in bromophenol blue free-Laemmli lysis buffer containing 0.125M Tris-HCl (pH 6.8), 4% (w/v) SDS, and 20% (v/v) glycerol and 10% (v/v) 2-mercaptoethanol. Cells were then boiled for 15 minutes at 95°C to denature proteins. To determine the quantity of protein in the lysates obtained, a protein assay (Bio-Rad, 500-0116) was performed following the manufacturer’s protocol, wherein standards were prepared by serial dilutions of bovine serum albumin (BSA) diluted in bromophenol blue free-Laemmli lysis buffer. A pre-calculated volume of sample containing 30µg of protein was loaded onto on Tris/glycine SDS-polyacrylamide gels alongside 5µL of marker ladder (Precision Plus Protein™, Bio-Rad, Hertfordshire, UK). The proteins on gels were then transferred onto Amersham Hybond-P PVDF membranes (GE Healthcare, Life science, UK) for 60 minutes at 100V at 4°C. The membranes were then blocked with TBS-Tween-20 (TBST) containing 5% (w/v) Marvel milk powder under a constant agitation for 1 hour at room temperature. After being thoroughly washed, membranes were blotted with primary antibodies at a concentration recommended by the manufacturer’s instructions (rabbit anti-human WT1 antibody, Abcam, Ab89901) and (rabbit anti-human DDX43 antibody, Sigma-Aldrich, HPA031381) and kept rocking overnight at 4°C. The next day, the membrane was washed 5 times with TBST, for 5 minutes each, and incubated in horseradish peroxidase (HRP)-conjugated secondary antibodies; HRP-conjugated anti-rabbit IgG antibody (Cell Signaling Technology, 70745, 1:1000) and HRP-conjugated anti-mouse IgG (Cell Signaling Technology, 70765). At the same time, Precision Protein™ Strep-Tactin™ HRP-conjugate, (Bio-Rad, 161-0380) was also added. Membranes were kept under a constant agitation for 2 hours at room temperature. After giving an additional five washes, membranes were developed using Clarity™ Western ECL Substrate (Bio-Rad, 170-5061) and luminescence was detected by G:BOX XT4: Chemiluminescence and Fluorescence Imaging System (Syngene). For each sample, mouse anti β-actin was used as a loading control (Sigma-Aldrich, A5441).

### Phenotypic Analysis of Target Cell Lines

Surface staining was performed to characterise target cells phenotypically to detect HLA-A2 expression using an (APC-conjugated anti-human HLA-A2 antibody (BioLegend, 343308, Clone: BB7.2). Phenotypic analysis was also performed to assess the success of HHDII gene transfection using an FITC-conjugated anti-human β2m antibody (Sigma-Aldrich, SAB4700012, Clone: β2M-01). Cells were incubated with PI immediately prior to running the samples. At the end of the assay, cells were re-suspended in Beckman Coulter Isoton II™ diluent and analysed immediately on the Beckman Coulter Gallios™ flow cytometer.

### DNA Vaccine-Bullet Preparation

Expression vectors encoding HAGE- and WT1-ImmunoBody^®^ (28) were coated onto 1.0μm gold microcarriers, as per the manufacturer’s instructions. In brief, 200μL of 0.05M spermidine containing 16.6mg of gold was mixed with 36μg of the ImmunoBody^®^ DNA. After giving the sample a short sonication, 200μL of 1M CaCl_2_ was then added in a dropwise manner to the mixture while vertexing, and then the DNA-gold complex was kept for 10 minutes at the room temperature. The pellet was then re-suspended in 2mL of 0.025mg/mL Polyvinylpyrrolidone after being washed twice with anhydrous ethanol. After this, and while the tube was sonicating, the sample was syringed into dried Tefzel™ tubing and kept standing in a Tubing Prep Station for five minutes. Without disturbing the gold, the ethanol was then gradually taken off using a syringe. Nitrogen gas was turned on while the tube was kept spinning for 7-10 minutes inside the station. When completely dried out, the tube was detached from the station and cut by a guillotine. Bullets were kept at 4°C until used.

### Immunisation Programme

HHDII/DR1 mice were immunised with DNA bullet containing 1µg of either HAGE DNA ImmunoBody^®^ which encodes the HAGE 30-mer sequence [QTGTGKTLCYLMPGFIHLVLQPSLKGQRNR] or WT1 ImmunoBody^®^ which encodes the WT1 15-mer sequence [VRDLNALLPAVPSLG] or both, administered by gene gun device on Day 1, 7 and 14. On Day 21, mice were culled and splenocytes were immediately harvested and used in an IFN-γ Enzyme-Linked ImmunoSpot (ELISpot) assay.

### *Ex Vivo* IFN-γ ELISpot Assay

The immunogenicity of either HAGE or WT1 peptides was determined using splenocytes harvested from immunised mice in an *ex vivo* IFN-γ ELISpot assay (ELISpot kit for mouse IFN-γ/MABTECH, 3321-2A). The assay was performed as previously described (29). Briefly 0.5x10^6^ of freshly isolated splenocytes were seeded per well in triplicates into the pre-coated IFN-γ ELISpot plate along with 1μg/mL of either HLA class I or 10μg/mL of HLA class II HAGE/WT1 derived peptides, cells were then incubated at 37°C in a 5% (v/v) CO_2_ incubator for 48 hours. The plates were then washed and biotinylated detection antibody against mouse IFN-γ was then added and incubated for 2 hours at room temperature. Plates were then washed followed by the addition of alkaline phosphatase (AP)-conjugated streptavidin, after which they were incubated for 60 minutes at room temperature. Plates were washed, development solution (BCIP/NBT, Bio-Rad) added left in dark at room temperature until spots could be seen. Once spots developed, the reaction was stopped by rinsing the plates under tap water. Plates were then left to dry and the spots were quantified using an ELISpot plate reader (Cellular Technology Limited). Staphylococcal enterotoxin-B (SEB), was used as a positive control, and unstimulated splenocytes (cells alone) were used as a negative control for every ELISpot assay. Animals were scored as having a positive reaction when the number of spots in the cells alone wells did not reach more than 20 spots and when the response in the peptide containing wells were at least twice that of standard deviation of the mean of the control wells.

### Chromium (^51^Cr) Release Cytotoxicity Assay

Splenocytes derived from vaccinated mice were cultured for 7 days with mitomycin-C treated peptide pulsed LPS blast cells as previously written (29). Briefly, LPS blast cells were prepared using splenocytes extracted from naïve mice and stimulated with LPS and dextran sulphate in a T75 flask. After 48 hours, cells from LPS blasts were harvested and treated with mitomycin-C. Following the incubation, cells were washed and counted and incubated with immunogenic HLA class I peptides, as determined by the *ex vivo* IFN-γ ELISpot assay for 90 minutes at 37°C. The peptide-pulsed LPS cells were then washed and co-cultured with freshly isolated splenocytes from immunised mice at a ratio (1) LPS (5): splenocytes in the presence of β-mercaptoethanol and murine IL-2 (mIL-2, Sigma-Aldrich, I0523, 50U/mL) at 37°C for 6 days. After incubation, LPS blast/T cells were then harvested, washed and counted to be ready for co-culture with ^51^Cr-labelled target cells.

Target cells were labelled with 1.85Mbq of ^51^Cr in a water bath at 37°C for one hour. Cells were then washed twice and rested for another hour at 37°C in 1mL of medium. Meanwhile, *in vitro* stimulated T cells were harvested and counted. Effector cells and target cells were plated in a 96-well rounded-bottom plate at a final volume of 200µL at different effector: target ratios of 100:1, 50:1, 25:1 and 12.5:1. Maximum and spontaneous release were set up in 4 replicates using 1% (w/v) SDS and plain medium, respectively. After 24 hours of co-culture of the effector and target cells at 37°C in 5% (v/v) CO_2_ incubator, 50μL supernatants were transferred to 96-well Luma plates to be dried on the plate. The ^51^Cr release was measured using a TopCount Microplate scintillation counter (beta scintillation counting) and the percentage of specific cytotoxicity was calculated using the following equation:

$$Percentage\;of\;cytotoxicity=\;\frac{\left(Experimental\;release-\;Spontaneous\;release\right)}{\left(Maximum\;release-\;Spontaneous\;release\right)}$$

### *In Vitro* Peptide Re-Stimulation (IVS) of the Murine Splenocytes

Fresh splenocytes isolated from vaccinated animals were stimulated with either HAGE 30-mer long peptides, WT1 15-mer long peptides or both, at a concentration of 1μg/mL in the presence of β-mercaptoethanol and 50U/mL mIL-2 for 7 days at 37°C in a 5% (v/v) CO_2_ incubator. Cells were then washed and counted using a NucleoCounter™ (Chemometec).

### Tumour Model

In the prophylactic setting, mice were immunised with 1µg of gold-coated HAGE-ImmunoBody^®^ DNA in one flank and 1µg of gold-coated WT1-ImmunoBody^®^ DNA into the contralateral flank simultaneously once, on 3 consecutive weeks (Day-1, -7 and -14). On Day-21, mice received 0.75x10^6^_h_B16/HAGE^+^/Luc^+^. In the therapeutic setting, 0.75x10^6^_h_B16/HAGE^+^/Luc^+^ cells were first implanted and the next day mice received the double vaccine which was then followed by two additional injections 7- and 14 days post tumour implantation. A control group of mice received the same dose of tumour cells, but no vaccines. Animals were monitored twice weekly and tumour growth assessed by callipers until a palpable tumour was detected. Mice were then monitored twice weekly by *in vivo* imaging until termination. For each imaging session, luciferin was administered intraperitoneally at 150mg/kg, and anaesthesia induction began 10 minutes afterwards. All mice were anaesthetised with an appropriate concentration of isoflurane and imaged using the IVIS Lumina III system (Perkin Elmer). Mice were sacrificed when the tumour volume reached a maximum of 1.2cm^2^ for the prophylactic group and 1.5 cm^2^ for the therapeutic group or show clinical signs.

### Processing of B16 Tumour Tissue

#### Tumour Volume and Weight

Once threshold endpoints were reached, tumours were dissected, weighed and scaled. Tumour volume was measured applying the formula: Π/6 (length X width^2^). Approximatively 1g of tumour was excised and enzymatically digested to study tumour-infiltrating lymphocytes (TILs), the remaining tumour was snap-frozen in liquid nitrogen.

#### Isolation of Tumour Infiltrating Lymphocytes (TILs)

Briefly, 1g of tumour tissue was excised from B16 tumours, chopped into small pieces using scissors and incubated with 5mL of RPMI containing 1mg/mL of collagenase-I (Sigma-Aldrich, C0130). The mixture was kept shaking at 37°C for 30 minutes to help tumour dissociation. Thereafter, the suspensions were filtered through a 70-μm filter, washed with PBS to remove the dissociation medium and re-suspended in 5mL of T cell medium. Cells (1x10^6^) were then stained for CD8 (APC/Cy7™-conjugated anti-mouse CD8a, BioLegend, 100714, Clone: 53-6.7), CD4 (Alexa-Fluor™ 700-conjugated anti-mouse CD4, BioLegend, 100430, Clone: GK1.5), CD3 (Brilliant Violet™ 421-conjugated anti-mouse CD3, 100228, Clone: 17A2) and PD-1 (APC-conjugated anti-mouse CD279 (PD-1), 135210, Clone: 29F.1 A12), as well as ZOMBIE™ or LIVE/DEAD™ Yellow™ viability stains to exclude dead cells. TILs were then analysed by a flow cytometry.

## Statistical Analysis

Data were analysed with GraphPad Prism7 software. The Mann-Whitney U-test was used for comparisons of two non-parametric datasets and one-way ANOVA or two-way ANOVA tests with *post hoc* testing using Tukey’s multiple comparison were used for multigroup comparisons. For tumour challenge studies, the survival proportion was evaluated by both the Gehan-Breslow-Wilcoxon test and the log-rank test. For each test, P-value (probability value) was calculated, and was found to be significant if *p-value=<0.05, very significant if **p-value=<0.01 and highly significant in case ***p-value=<0.001 or ****p-value =<0.0001.

## Results

### Prediction of Peptide Binding to HLA-A2 and HLA-DR1

The immunogenic region of the HAGE antigen was previously identified in our group using combination approaches of matrix-screening method of overlapping peptides and reverse immunology. It has been found that a 30-mer peptide sequence [QTGTGKTLCYLMPGFIHLVLQPSLKGQRNR]/[position: 286-316] derived from the HAGE protein is associated with high immunogenicity, as detected using an *ex-vivo* IFN-γ ELISpot assay (22). From that sequence, various short peptides were predicted to bind to HLA class I (HLA-A2) and HLA class II (HLA-DR1) using the freely available software SYFPEITHI, **Tables 2A, B**. Only HLA-A2 and HLA-DR1 restricted peptides were selected to be used in the HLA-A2/DR1 double transgenic mice (HHDII/DR1). Similarly, a 15 amino acid HLA-DR1 binding peptide [VRDLNALLPAVPSLG] derived from the WT1 protein containing two known HLA-A2 9-mer (30) was initially assessed for its binding affinity using the SYFPEITHI database (**Tables 2C, D**).

**Table 2:** Class I and class II peptides sequence derived from the entire length of the HAGE and WT1 proteins.

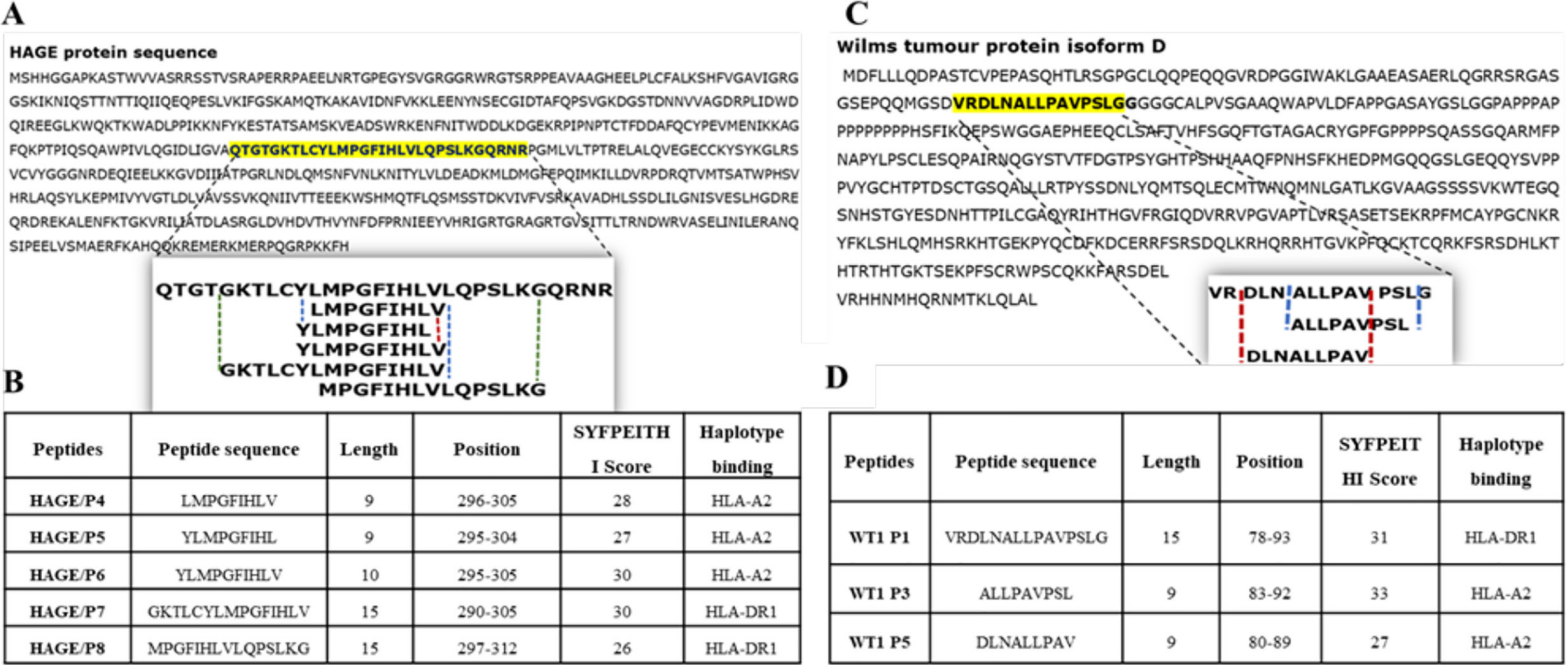	

The entire HAGE protein sequence of 648 amino acid length, from which the long 30-mer (yellow highlighted in A) and other short 9-15-mer peptides derived from the long 30-mer are shown in A&B. This length was fully copied from NCBI website/FASTA, available at: https://www.ncbi.nlm.nih.gov/protein/Q9NXZ2.2?report=fasta. Whereas the entire WT1 protein sequence of 522 amino acid length (Isoform-D), from which the long 15-mer (yellow highlighted in C) and the two others short peptide derivatives are illustrated in C&D. This length was fully copied from NCBI website/FASTA available at: https://www.ncbi.nlm.nih.gov/protein/NP_077744.4?report=fasta.

### *In Vitro* Peptide-HLA-A*0201 Molecule Affinity and Stability

**Figure 1:**
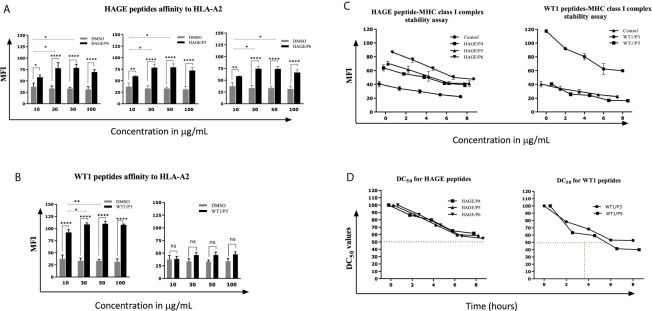
*In vitro* determination of the HAGE and WT1 peptides-HLA-A*0201 molecule affinity and stability. The affinity of class I HAGE peptides (HAGE/P4, HAGE/P5 and HAGE/P6) shown in **(A)** and WT1 peptides (WT1/P3 and WT/P5) shown in **(B)** to bind HLA-A2 molecules in comparison to controls (DMSO-treated cells) was determined using the T2 binding assay. T2 cells were incubated with 10, 30, 50 and 100µg/mL peptide in serum-free media containing 3µg/mL human β2m overnight in a 26°C incubator. Cells were stained with an anti-HLA-A2 antibody and propidium iodide (PI), and then immediately analysed using a flow cytometer. Values are expressed as the mean ± SD of three independent experiments and the level of significance was determined using Two Way ANOVA, followed by Tukey’s multiple comparisons test. Results indicate that all peptides were strong binders to MHC class I with the exception of WT1/P5. The binding stability (measured by MFI) of class I HAGE peptides and WT1 peptides shown in **(C)** in comparison to cells that were incubated with the same concentration of DMSO. DC_50_ for these peptides is shown in **(D)**. T2 cells in serum-free medium containing human β2m at 3µg/mL were incubated with 50µg/mL of the respective peptides overnight and assessed in a time course analysis for HLA-A2 expression using a flow cytometer. Values are expressed as the mean ± SD of two independent experiments. All peptide-HLA complexes studied are stable on the T2 cells surface as their DC_50_ values were > 8 hours, apart from the WT1/P5- HLA-A2 complex which shows poor stability as there was a drop in DC_50_ value earlier between 5-6 hours. *p-value=<0.05, significant; **p-value=<0.01, very significant and ****p-value=<0.0001, highly significant, ns, not significant.

Several factors influence the capacity of peptide-MHC complexes to stimulate T cells, of which the primary two are the affinity of peptides for the MHC molecules and the cell surface stability of the peptide-MHC complex (31, 32). Although scoring of peptide binding to HLA-A2 *via* the computer algorithm predictions is useful, its accuracy is less than 70–80% (33). Therefore, the strength and stability of the interaction between the respective HAGE and WT1 class I peptides and the HLA-A2 molecules were assessed experimentally using peptide-HLA-A2 T2 binding and stability assays, respectively. Overall, data in (**Figure 1**) (A, B) demonstrate that the intensity of HLA-A2 expression as measured by Mean Fluorescence Intensity (MFI) progressively increases in a dose-dependent manner in comparison with the control (MFI produced by cells that were treated with matched doses of DMSO vehicle control), indicating a successful peptide-MHC engagement, apart from WT1/P5 which did not increase the MFI, thereby reflecting its poor binding affinity toward HLA-A2 molecules. In addition, the Fluorescence Intensity Ratio (FIR) was calculated for each peptide concentration using the MFI of HLA-A2 expressed by T2 cells incubated with peptides and the MFI of HLA-A2 expressed by T2 cells that were incubated in the absence of peptide, but with the same concentrations of DMSO as background level of HLA-A2 molecules present on the surface of T2 cells. A summary of the results is shown in **Table 3**. Peptides with FIR=1 were categorised as being non-binders, 1<FIR ≤ 1.5 as weak binders, 1.5<FIR<2 as moderate binders and 2≤FIR as strong binders. All HAGE peptides demonstrated strong binding affinity towards HLA-A2 molecules, from 30μg/mL onwards (**Figure 1A** and **Table 3**). WT1 peptide, P3, was found to be a strong binder even at 10μg/mL, whereas WT1/P5 exhibited the least binding affinity with a score of 1 (non-binder) at 10 µg/mL, and only weak binding capability (1.4 to 1.5) at the higher concentrations (**Figure 1B** and **Table 3**).

**Table 3:** Categorisation of the binding affinity of class I HAGE- and WT1-derived peptides toward HLA-A2 molecules, as assessed using the T2 binding assay.

Peptides	FIR obtained from various HAGE and WT1 peptide doses			
10µg/mL	30µg/mL	50µg/mL	100µg/mL
HAGE/P4	1.8 (moderate)	2.3 (strong)	2.4 (strong)	2.3 (strong)
HAGE/P5	1.9 (moderate)	2.4 (strong)	2.4 (strong)	2.3 (strong)
HAGE/P6	1.8 (moderate)	2.3 (strong)	2.3 (strong)	2.2 (strong)
WT1/P3	2.6 (strong)	3.3 (very strong)	3.4 (very strong)	3.5 (very strong)
WT1/P5	1.0 (non)	1.4 (weak)	1.4 (weak)	1.5 (weak)

Serial doses were used to assess peptides binding affinity toward HLA-A using flow cytometry, peptides were then categorised for being a strong, moderate, and weak HLA-A2 binder according to Fluorescence Intensity Ratio (FIR).

The persistence of the peptide-HLA class I complex on the cell surface was assessed in a time course manner using a Brefeldin A (BFA) assay (**Figures 1C, D**). BFA has a trap effect by obstructing anterograde transport of vesicles from the ER to the Golgi apparatus, thereby preventing peptide-MHC proteins from being transported to the cell surface. Results in **Figures 1C** show that there is a steady, time-dependent reduction in the level of MFI in all complexes within the first hours of study, but that the reduction stopped after 6 hours. With regards of the DC_50_ value, all HAGE and WT1/P3-HLA-A2 complexes on the T2 cell surface were stable as their DC_50_ values were all more than 8 hours (**Figure 1D**). However, WT1/P5-HLA-A2 complex exhibited a poor stability, as DC_50_ value fell before 6 hours.

### Immunogenicity of HAGE- and WT1-ImmunoBody^®^ Vaccines

**Figure 2:**
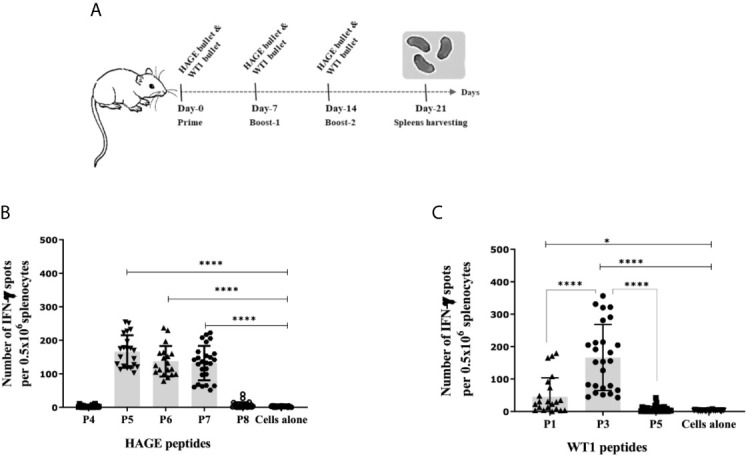
Immunogenicity of HAGE- and WT1-ImmunoBody® vaccines, as determined using the IFN-γ ELISpot assay. Schematic presentation of ImmunoBody^®^ immunisation regime is demonstrated in **(A)** where a prime-boost strategy was applied to assess the immunogenicity of HAGE-and WT1- ImmunoBody^®^ administered in separate sets of mice. In this programme, each mouse received a total of three injections at 7-day intervals and spleens were then harvested on Day-21 for IFN-γ ELISpot assay. The level of IFN-γ produced by fresh 0.5x10^6^ splenocytes harvested from a group of mice immunised and boosted with HAGE-ImmunoBody^®^ **(B)** and WT1- ImmunoBody^®^ vaccine **(C)** were assessed using the ELISpot assay. Values are expressed as the mean ± SD of at least 8 independent experiments (3 mice/group) and the level of significance was assessed using One Way ANOVA followed by Dunnett’s multiple comparisons test. Results in **(B)** demonstrate the potent immunogenicity of the two class I peptides; HAGE/P5 and HAGE/P6 and one class II; HAGE/P7 in comparison to cells alone, whereas the class I HAGE/P4 and class II HAGE/P8 are shown to be poorly immunogenic. Results in **(C)** demonstrate the potent immunogenicity of WT1/P3, mild immunogenicity of WT1/P1 and poor immunogenicity of WT1/P5 in comparison with the absence of peptide ‘cells alone’. *p-value=<0.05, significant and ****p-value=<0.0001, highly significant.

To determine the immunogenicity of the HAGE (30mer) and WT1 (15mer) sequences and whether the predicted HLA-A2 and HLA-DR1 derived from these sequences were endogenously processed, HHDII/DR1 mice were immunised with either HAGE-ImmunoBody^®^ or WT1-ImmunoBody^®^ constructs on Day- 0, -7 and -14 (**Figure 2A**). On Day 21, animals were culled, and their respective spleens harvested and assessed in an *ex-vivo* IFNγ ELISpot assay. Results in **Figure 2B** confirms the immunogenicity of two class I peptides (HAGE/P5 and HAGE/P6) and one class II (HAGE/P7) in comparison to cell alone (cells that did not receive any peptide) as reflected by the high number of IFN-γ producing cells (*P-value<0.0001, n=3)*. Whereas, HAGE/P4 and HAGE/P8 peptides did not produce any IFN-γ, indicating that these peptides were not endogenously produced and presented to T cells *in vivo*. WT1-specific responses induced by a novel WT1 DNA ImmunoBody**^®^** vaccine was also assessed and the results in **Figure 2C** demonstrate the immunogenicity of WT1/P1 and WT1/P3, and the poor immunogenicity of WT1/P5.

### *In Vitro* Recognition and Killing of CML Targets Expressing HLA-A2, HAGE and WT1 Antigens by Dual Vaccine-Induced T Cells

**Figure 3:**
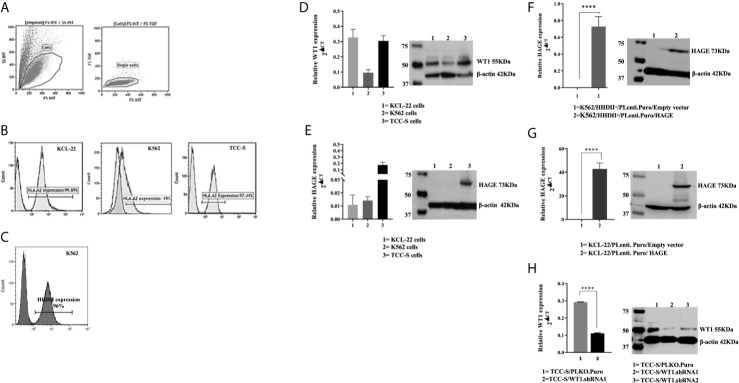
HLA-A2, HAGE and WT1 expression in target cells: **(A)** Representative gating strategy used for the analysis of the surface expression of HLA-A2 on targets, as assessed by flow cytometry. Live cells were first gated according to their Side Scatter and Forward Scatter profile, and then single cells were selected, avoiding doublets. **(B)** The level of surface HLA-A2 expression was determined on basis of fluorescence intensity depicted by a shift to the right of the histogram in comparison to unstained samples. **(C)** Overlay histogram illustrates HLA-A2 surface expression on the targets in comparison to a negative control (non-stained sample). The constitutive expression of WT1 and HAGE by KCL-22, K562 and TCC-S cell lines using RT-qPCR and Western blot shown in **(D, E)**. HAGE is transcribed at low levels in KCL-22 and K562 cells, but protein level are high. In contrast, TCC-S cells have constitutively high HAGE transcript and protein levels. WT1 was expressed by all cells studied as detectable by transcription and and protein levels. The Success of transfection; knock-in and knockdown, seen in **(F–H)** Values of PCR are expressed as the mean ± SD of two independent experiments. The doublet on WT1 seen in the Western blot might represent WT1 isoforms resulting from an alternative splicing event. ****p-value=<0.0001, highly significant.

K562, KCL-22 and TCC-S CML cell line-derived targets were assessed for HLA-A2 expression. Histograms in **Figure 3B** shows that that only a small percentage of K562 cells naturally express HLA-A2, whereas a high percentage of KCL-22 and TCC-S cells are HLA-A2 positive (**Figures 3A, B**). Therefore, K562 cells were electroporated with PcDNA-3.1/HHDII construct encoding the chimeric HLA-A2 gene (HHDII). **Figure 3C** demonstrates the success of HHDII transfection of K562 cells.

The constitutive expression at mRNA and protein levels of both HAGE and WT1 was then determined in all CML targets using RT-qPCR and Western blotting (**Figures 3D, E**). All cell lines expressed WT1 mRNA and protein, whereas HAGE mRNA and protein expression was only detected in TCC-S cells. Therefore, both K562/HHDII^+^ and KCL-22 cells were then transduced with PLenti-Puro/HAGE plasmid construct, followed by antibiotic selection using puromycin to maintain HAGE expression in addition to G418, for K562/HHDII, to maintain HHDII gene expression. Post selection, the success of the transduction was demonstrated at mRNA and protein levels using RT-qPCR and Western blot (**Figures 3F, G**).

TCC-S cells were found to express HLA-A2, WT1 and HAGE proteins. It was expected that knockdown of WT1 in these cells could establish a negative control for WT1 expressing TCC-S (wild-type) upon assessing the responsiveness of WT1-specific CTLs in the cytotoxicity assay. Data in **Figure 3H** demonstrate the outcome of WT1 knockdown in TCC-S at protein level using two WT1.shRNA sequences in comparison with cells that were transfected with the lentiviral carrying the empty vector (PLKO.1.Puro), as a control vector. Although both sequences of shRNA were successful in significantly inhibiting WT1 protein expression, the WT1.shRNA.1 appeared to be more efficient than WT1.shRNA.2, and therefore the WT1.shRNA.1 clone was used in the cytotoxicity assay. All the modified cell types were periodically checked for the maintenance of the transduced genes.

Once all the target cells had successfully been “engineered” to express the correct HLA-A haplotype and/or antigens the ability of the vaccine induced cells to recognised and kill them was assessed. Briefly, splenocytes harvested from animals immunised with either HAGE-ImmunoBody^®^ alone, WT1-Immunobody^®^ alone or both were co-cultured *in vitro* with mitomycin-C treated and peptide-pulsed LPS blasts at ratio of 1:5 (LPS blast: T cell) for 6 days. Cells were then plated with ^51^Cr labelled K562 cells at various target: effector ratios (1:100, 1:50, 1:25, and 1:12.5) for 24 hours at 37°C. Supernatants were then transferred onto Luma plates and read using a TopCount beta scintillation counter.

**Figure 4:**
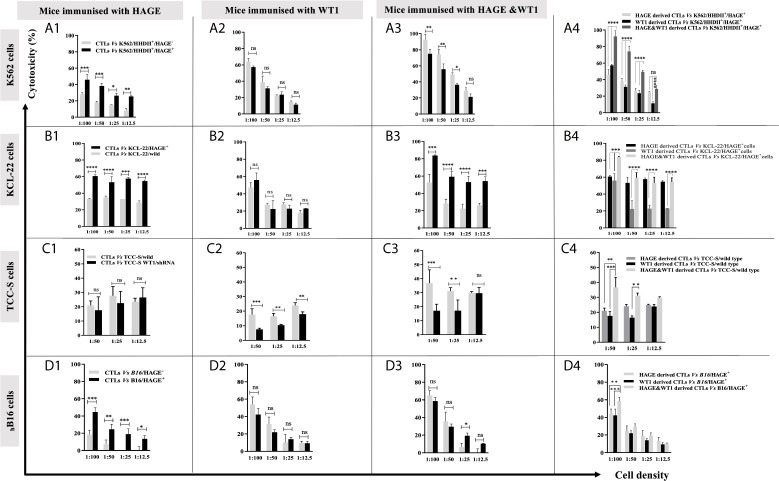
Specific *in vitro* killing of target cells by vaccine induced CTLs, as assessed by 51Cr release assay. Splenocytes from mice immunised with HAGE, WT1 and combined, were co-cultured in vitro with mitomycin-C treated and peptide-pulsed LPS T cells for 6 days and then plated with ^51^Cr labelled-targets at various ratios for 24 hours at 37°C, followed by measuring ^51^Cr in the medium. Values are expressed as the mean ± SD of three independent experiments (3 mice/group) and the level of differences between groups were assessed using Two Way ANOVA followed by Tukey’s multiple comparisons test. Results demonstrate that the percentage of lysis generated from combined vaccine is higher than the individual vaccines. P*-value < 0.05, **p-value < 0.01, ***p-value < 0.001, ****P-value < 0.0001 versus the corresponding control group. NS, not significant.

**Figure 4** shows that *in vitro* expanded cells from all vaccinated mice were able to specifically recognise and kill all the target cells expressing the antigen against which the mice were vaccinated, **Figure 4** (A1, A2) (B1, B2) (C1, C2). Moreover, cells derived from the mice immunised with both HAGE- and WT1-ImmunoBody^®^ vaccines could consistently kill significantly more target cells expressing both antigens indicative of a synergistic effect achieved with the combined vaccines **Figure 4** (A3, A4) (B3, B4) (C3, C4) *(P-value<0.0001, n=3)* in almost all ratios used.

In our previous studies, the B16/murine MHC knockout/HHDII+/DR1+ murine melanoma cell line was shown to constitutively express the murine WT1 protein (data not shown). Herein, these cells (with/without HAGE) were used as targets to assess the capability of HAGE- and WT1-ImmunoBody^®^ vaccines derived cells to provoke *in vitro* killing activity. **Figure 4D** highlights the significant difference in the lysis produced by each indicated group against these cells. Here again, a statistically significant difference was found between the killing of B16 cells expressing HAGE (almost 50%) and HAGE-negative B16 (20%) at 1:100 ratio (shown in D1), whereas no differences in killing were found against these two targets when the effector cells used were derived from mice immunised with only WT1 (shown in D2), since both express murine WT1. This indicates that the WT1 peptide used in this study was endogenously processed naturally and presented on the surface of the B16 cells and was recognised by cytotoxic T lymphocytes (CTLs). Again, as for the other targets, a significantly higher percentage of B16/HAGE^+^ cells was killed when the CTLs used were derived from mice immunised with the combined approach (**Figure 4D**, at almost 60%) than when they were derived from mice immunised with either HAGE or WT1 vaccine individually (50% and 45%, respectively). This difference is shown to be a statistically significance at 1:100 (target: effector ratio) (*P-value=0.0032, n=3* and *P-value=0.0002, n=3*) in HAGE and WT1 groups, respectively.

Overall, these findings demonstrate that the sequences contain within HAGE- and WT1-ImmunoBody^®^ derived vaccines either individually or in combination was endogenously processed and displayed on the surface of antigen presenting cells (APCs) in association with HLA-A2 molecules leading to the development of professional CTLs which are able of specifically recognising and killing antigen-expressing target cells. They also indicate that the combination approach improves CTL responses.

### Efficacy of the Combined HAGE/WT1 ImmunoBody^®^ Vaccines in HHDII/DR1 Mice Bearing the Aggressive _h_B16/HAGE^+^/Luc^+^ Tumour

**Figure 5:**
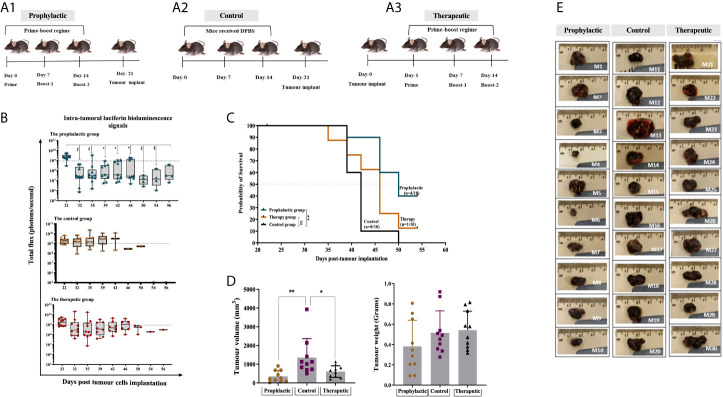
Combined HAGE/WT1 ImmunoBody® vaccines significantly delay tumour growth in tumour challenge experiment. Schematic representation of the in vivo experimental design is shown in **(A)** where three groups of mice were used in this study using prime- boost regime. In the prophylactic setting, mice were received the vaccine then challenged with hB16/HAGE^+^/Luc^+^ cells on Day-21, whereas, in the therapeutic setting, tumour cells were implanted, followed by vaccination in the determined days. The control group of mice received DPBS instead of the vaccine. Intra-tumoural luciferin bioluminescent signals in the vaccinated HHDII/DR1 mice bearing the aggressive hB16/HAGE^+^/Luc^+^ tumour in comparison to the control are shown in **(B)**. The figure demonstrates the prophylactic and therapeutic efficacy of the combined HAGE/WT1 ImmunoBody® vaccines, wherein luciferin bioluminescent signals were detected in all groups as a total flux measured by photons/second. Data shows that the total flux in the prophylactic group and therapeutic declined in comparison with the control, and that both treatment settings led to prolong mice survival rate. Most mice from the control group were culled after Day-39 post transplantation session whereas mice in the vaccinated groups survive while they were holding small size tumour. Post-culled hB16/HAGE^+^/Luc^+^ tumour weight and volume are shown in **(C, D)**. For each indicated group, tumour weight in grams and tumour volume in mm3 were determined, and post-culled tumour images highlighted the tumour size shown in **(D)**. Mice were euthanized when tumour size reached endpoint threshold (12 mm2 for the prophylactic setting and 15 mm2 for the therapeutic setting). **(E)** Data in these graphs are expressed as the mean ± SD, and the level of the significance was determined using one-way ANOVA analysis followed by Dunnett’s multiple comparisons test. Survival analysis demonstrates the antitumor efficacy of the combined HAGE/WT1 ImmunoBody® vaccines in vaccinated tumour bearing mice. Results shows that the combined HAGE/WT1 vaccines is significantly protective in the prophylactic group in comparison to the control group at **P-value<0.01, but not in the therapeutic setting. The significance of the difference was evaluated by both Gehan-Breslow-Wilcoxon test and log-rank test. The total number of mice is 30, (10/group). *p-value=<0.05, significant; **p-value=<0.01, very significant, ns, not significant.

The efficacy of the combined HAGE/WT1 ImmunoBody^®^ vaccines was then tested in both prophylactic and therapeutic settings in female HHDII/DR1 mice (n=10/group) using the aggressive _h_B16/HAGE^+^/Luc^+^ tumour. In the prophylactic setting, mice were first immunised with both HAGE- and WT1-ImmunoBody^®^ vaccines 3 times, a week apart, and on Day 21 mice were then challenged with subcutaneous injection of _h_B16/HAGE^+^/Luc^+^ cells, as shown in **Figure 5A**1. In the therapeutic setting, mice were first implanted with the same dose of _h_B16/HAGE^+^/Luc^+^ cells, followed by immunisation the next day, and then received two more injections of the dual HAGE/WT1 ImmunoBody^®^ vaccines 7 days apart. The control group received no vaccine (**Figures 5A2, A3**).

Details of bioluminescence images and mice culling per session are shown in **Supplementary Figure 2**. Mice that did not receive the vaccines had to be sacrificed as early as Day 39 post-implantation due to tumour size endpoints, whereas tumour growth was delayed in mice that combined vaccines in comparison to the controls (**Figure 5**). Indeed, at around day-46 post-implantation, the number of surviving mice was 8/10 (80%) for the prophylactic group and 6/10 (60%) for the therapeutic group, in comparison with only 1/10 (10%) for the control group. Tumour growth, as measure by the total luminescence flux for each group was recorded and plotted in **Figure 5B**. Eventually, 4 mice from prophylactic group (Mouse-6, Mouse-7, Mouse-9 and Mouse-10) and one mouse from the therapeutic group (Mouse-24) were the last mice to be culled. The study was then terminated on Day 56 post-implantation before the tumours reached their end point size. This was purposely done in order to be able to perform immune characterisation of the tumour infiltrating lymphocytes extracted from the growing tumour and performed functional assay on splenocytes extracted from these mice.

Interestingly, although the total luminescence flux signals, as expected, were very similar on Day 22 (one day post-transplant), 10 days later, a statistically difference between the size of the tumours for the prophylactic group and those for the control was detected. Thereafter, the size of the tumour in the prophylactic group and for the therapeutic group remained below the 1x10^9^ threshold for the majority of the tumours. However, by Day 39, tumours in a distinct group consisting of 4 mice separated from the rest of the prophylactic group, continued to grow until they had to be culled on Day 46. In the future, it would be important to re-immunise animals showing such a trend before their tumour became too large. It is also evident from the data that for 7 out of the 10 mice that received the vaccine after tumour implantation, the tumour first regressed below the 1x10^9^ threshold, whereas the size of the tumours from the control group was, for the majority, above or very near this value (with the exception of one mouse), but then by Day-39 these increased again. This demonstrates the ability of the combined vaccines to delay the growth of the tumour, but there would likely be a requirement for further vaccination on Day-39 or additional interventions, such as checkpoint inhibitor. The survival proportion in **Figure 5C** clearly demonstrates that the combined HAGE/WT1 ImmunoBody^®^ vaccines were able to significantly delay the aggressive growth of B16 melanoma cells and increases the overall survival in the prophylactic setting in comparison to the control at **P-value<0.01. However, this level of efficacy was not shown in the therapeutic group.

Tumour volume and weight were measured as soon as animals were culled. Data in **Figure 5D** demonstrates that there was a significance difference in the tumour volume in the prophylactic and therapeutic group compared to the control (*P-value =0.003, n=10* and *P-value= 0.0271, n=10*), respectively, which indicates a shrinkage of the tumour, probably due to the activation of anti-B16 CTLs.

### The Combined HAGE/WT1 ImmunoBody^®^ Vaccine Provokes *In Vivo* Anti-Tumour Effector T Cell Function in HHDII/DR1 Mice Bearing _h_B16/HAGE^+^/Luc^+^ Tumour

Until Day 56 post-implantation, all mice were sacrificed due to tumour size reaching the endpoint threshold, except for Mouse-6, Mouse-7, Mouse-9 and Mouse-10 from the prophylactic group and Mouse-24 from the therapeutic group. However, it was decided to terminate the study to enable the analysis of TILs and perform functional assays, such as IFN-γ ELISpot.

**Figure 6:**
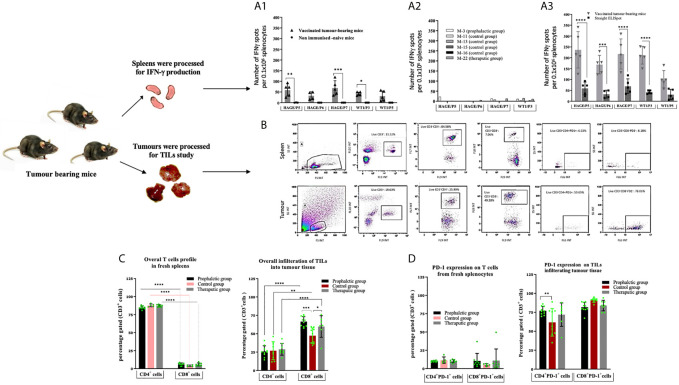
Number of IFN-gamma symbol producing cells can be further expanded *in vitro*. Tumour induced PD-1 expression on T-cells. *Ex vivo* IFN-gamma symbol ELISpot analysis on splenocytes derived from HHDII/DR1 tumour bearing mice vaccinated by the combined HAGE/WT1 ImmunoBody® vaccines shown in **(A)**. Result of direct ELISpot assay on fresh splenocytes derived from the last surviving tumour-bearing mice from the prophylactic group (Mouse-6 ,Mouse-7 ,Mouse-9 and Mouse-10) and one mice from therapeutic group (Mouse-24) shown in **(A1)** versus samples from mice culled earlier shown in **(A2)**, cells were harvested and plated at a density of 0.5x106 cells/well and re-stimulated with 1µg/mL of short peptides for 48hours. Results demonstrate that IFN-gamma symbol is much greater in the surviving mice than the control. These splenocytes were also assessed in vitro after one-week stimulation using IVS assay, wherein, cells were incubated at a density of 0.1x106 cells/well with 1ug/mL short peptides shown in **(A3)**. Results reveal that there was a significance induction of IFN-gamm symbol secretion after 1-week IVS. Data plotted as an average of spots/mouse, expressed as the mean ± SD of all 5 mice and the level of the significance was assessed using two-way ANOVA. Cells were processed and incubated with anti-mouse FcR block at 1μg/test for 15 minutes at 4°C and then stained with surface antibody; anti-CD3, -CD4, -CD8, -PD-1 and LIVE/DEAD YellowTM stain for 30 minutes at 4°C, cells were then assessed by a flow cytometry. Overall profile of T cells derived from spleens and TILs harvested from vaccinated and non-vaccinated tumour-bearing mice were analysed according to the gating strategy shown in **(B)** and the results are presented in **(C)** (for the % of T-cells) and **(D)** (for the % of PD1+). Data are expressed as the means± SD of at around 10 mice/group and the level of significance were assessed by two-way ANOVA followed by Dunnett’s multiple comparisons test. Data demonstrate an inversed CD4:CD8 ratio derived from tumour in comparison to spleens and demonstrates PD-1 upregulation in the T cells derived from tumour tissues. *p-value=<0.05, significant; **p-value=<0.01, very significant; ***p-value=<0.001 and ****p-value=<0.0001, highly significant, ns, not significant.

The immune response generated in these mice was compared with that of the IFN-γ response assessed in a set of mice culled earlier in the study, (Mouse-11, Mouse-13, Mouse-15 and Mouse-16) from the control group and one mouse from the therapeutic group (Mouse-22). Interestingly, comparing data in **Figure 6A1** *versus* **A2**, IFN-γ production was significantly greater in the surviving mice than those which were culled early in the study due to their tumour size reaching the end point. Vaccinated mice which had to be sacrificed early in the study due to tumour size (such as mouse-22) lacked the development of a specific anti-tumour immune response and therefore could not maintain tumour growth in a similar pattern as the control. IFN-γ production from spleens of these mice was also assessed after 1week *in vitro* stimulation (IVS) using the IFN-γ ELISpot assay. As demonstrated in **Figure 6A3**, IFN-γ secretion was significantly increased after IVS in comparison with those obtained straight *ex-vivo* demonstrating that these cells can be further expanded *in vitro* after *in vivo* priming.

### Phenotypical Characterisation of T Cells and TILs From Mice Bearing _h_B16/HAGE^+^/Luc^+^ Tumour

The profile of tumour-infiltrating lymphocytes (TILs) was assessed and compared with T cells extracted from spleens from tumour bearing mice. After each culling, a small piece of tissue was immediately digested and stained with anti-mouse CD3, CD4, CD8a and CD279 PD-1 antibodies. Thereafter, samples were assessed by flow cytometry and analysed according to the gating strategy shown **Figure 6B**. **Figures 6C, D** demonstrates the expression pattern of CD3^+^, CD4^+^, CD8^+^ and PD-1 on TILs and T cells from freshly isolated splenocytes from tumour bearing mice in both treatment settings in comparison to the control group. From these data, the following points can be concluded; firstly, the majority of CD3^+^ T cells recruited in spleens were CD4^+^ cells, whereas the majority of CD3^+^ T cells in all tumours studied were CD8^+^ cells, indicating a reversal in the CD4^+^/CD8^+^ ratio of TILs in comparison with those derived from the spleens. Although this trend was observed regardless of whether the mice were vaccinated, the percentage of CD8^+^ T cells within the tumour of the prophylactic and therapeutic mice was significantly higher than the control at ***P-value= 0.0003 and *P-value= 0.0323, respectively, indicating that the combined vaccines were able to recruit more CD8^+^ cells in the vaccinated group in both the prophylactic and therapeutic settings. Secondly, more than 60% of CD4^+^ and CD8^+^ TILs expressed PD-1 compared to 5-10% in the spleens, suggesting that T cells of both types CD4^+^ and CD8^+^ in the tumour microenvironment have been jeopardised by immunosuppressive elements, PD-1, which might be induced by immune immunosuppressive cells in the hostile B16 microenvironment. Finally, the percentage of CD4^+^PD-1^+^ cells in the tumour was higher in the prophylactic group at **P-value= 0.0046 than the control.

## Discussion

Although the choice of antigen and peptide sequence remain crucial elements for the success of peptide-based vaccines, the adjuvant and/or delivery system and route of delivery used with these also play an important role in the intensity and duration of the response. It is now well established that long peptide vaccines are preferred to single CD8 epitopes due to their capacity to harbour multiple CD8^+^ and CD4^+^ T cell epitopes and bind to a wider range of HLA-haplotypes, thereby increasing their efficiency at generating durable anti-tumour responses (34, 35). In addition, the targeting of multiple antigens, and epitopes therein, reduces the risk of escape variants. HAGE and WT1, have both been shown to be immunogenic (36, 37), with the HAGE long 30-mer peptide sequence [QTGTGKTLCYLMPGFIHLVLQPSLKGQRNR] having been found to be immunogenic and to encompass several immunogenic class I and class II restricted epitopes that bind to HLA-A2 and HLA-DR1 haplotypes (23). The superiority of ImmunoBody^®^ over peptide immunisation has previously been demonstrated using several different antigens including HAGE (23, 28). Hence, the present study focused on the use of two ImmunoBody^®^ vaccines - HAGE-ImmunoBody^®^ and WT1- ImmunoBody^®^ vaccine. In a similar manner, a 15 amino acid peptide [VRDLNALLPAVPSLG] derived from the WT1 protein contained two known and previously published HLA-A2 9-mers, and was selected so the efficacy of the WT1 vaccine could be studied. Here the combination of both HAGE and WT1 HLA-A2 restricted epitopes were evaluated for their MHC binding affinity *in vitro* using a T2 binding assay. Results showed that all HAGE HLA-A2 restricted peptides; HAGE/P4, HAGE/P5, and HAGE/P6 were able to stabilise HLA-A2, as was reflected by the high FIRs which correlated with the SYFPEITHI software predication of high binders. Likewise, the binding affinity of WT1 peptides to HLA-A2 was experimentally studied. WT1/P3 was found to be a very strong HLA-A2 binder, which would reasonably be expected to activate WT-1 specific T cells *in vitro* and *in vivo*. On the other hand, although WT1/P5 has a predicted SYFPEITHI score of 27, it exhibited low binding capability to HLA-A2 which was not improved with higher peptides concentrations. It was therefore categorised as being a weak HLA-A2 binder. However, while a high peptide binding affinity to MHC is associated with high frequency peptide-MHC complex production, it does not necessarily mean that these complexes will induce potent T cell activation, as in some instances peptide-MHC complexes are relatively unstable and subject to rapid recycling and endocytosis, thereby hindering T cell recognition and subsequent killing. In addition, it has been reported that the peptide-MHC stability, more than the peptide-MHC binding affinity, is linked to T cell activation (38). It was therefore decided to study the stability of the respective peptide-MHC complex on T2 cells using DC_50_ as an indicator (the time required for a 50% reduction in the MFI), such that the higher the DC_50_ the more stable is the interaction between peptide and MHC. This study revealed that all HAGE-HLA and WT1-HLA complexes were associated with rapid dissociation within the first 6 hours of the experiment, as reflected by the drop in the MFI values, however this decline seemed to stop thereafter. As far as the DC_50_ value is concerned, the results indicated that all complexes were technically stable, as there was less than 50% reduction in MFI in comparison to time zero during the time range of the experiment (>8 hours), except for WT1/P5-HLA-A2 complex which showed poor stability as its DC_50_ value was between 5-6 hours. Collectively, the feasibility of using the candidate peptides for future experiments was validated, as they were associated with strong binding affinity toward HLA molecules as well as potential resistance to the dissociation, except for WT1/P5 which demonstrated low binding and early dissociation from HLA-A2 molecules.

The endogenous presentation of these peptide after vaccination of HHDII/DR1 mice with HAGE-ImmunoBody^®^ and WT1-ImmunoBody^®^ were then assessed using direct *ex vivo* IFN-γ ELISpot in order to determine the frequency of T cell responses. Class I HAGE/P5, HAGE/P6 and class II HAGE/P7 epitopes were all associated with high T cell responses. However, no specific IFN-γ response was detected for the class I peptide HAGE/P4, indicating this peptide may not be endogenously processed, despite being a strong and stable HLA-A2 binder. WT1/P3 epitope was found to elicit high number of IFN-γ producing cells in response to WT1-ImmunoBody^®^ vaccination while WT1/P5 peptide was not recognised by the vaccine-induced splenocytes as indicated by a lack of IFN-γ production.

The capability of HAGE/WT1-ImmunoBody^®^ induced T cells to recognise and kill HAGE and WT1 expressing leukaemia cells was then assessed using *in vitro* cytotoxicity assays. For this purpose, three CML cell lines were chosen, K562, KCL-22 and TCC-S cells. TCC-S and KCL-22 cells were found to express HLA-A2, unlike K562, therefore the latter was transfected with the chimeric HHDII construct to be compatible with HHDII/DR1 mice. In addition, as both K562 and KCL-22 cells do not express HAGE, they were transduced with the HAGE construct. All cell lines expressed WT1. TCC-S cells were the only cells found to express HAGE, WT1 and HLA-A2. Some of these cells were transfected with shRNA for WT1 to produce TCC-S-HAGE^+^/WT1^Low^ cells. Splenocytes derived from HAGE/WT1-ImmunoBody^®^ vaccinated animals were stimulated once *in vitro*. These splenocytes were able to recognise and kill targets in an antigen dependant manner. Interestingly, although the number of IFN-γ producing cells generated with either vaccine alone was similar to those generated when both were used simultaneously, the combined vaccine led to a significant increase in cell killing compared to either vaccines alone. Importantly, the level of HAGE protein detected in the transduced cell lines was similar to that of the naturally HAGE expressing TCC-S cells.

In the absence of an available murine CML model that can be used in the HLA transgenic mice we tested the efficacy of this combination in *in vivo* tumour challenge experiments, wherein a pre-defined dose of the modified _h_B16/HAGE^+^/Luc^+^ cells, which also endogenously express WT1, was injected subcutaneously as a “Proof-of-Concept” to form tumour. The combination vaccines were assessed in prophylactic and therapeutic trial. This melanoma-derived tumour model is obviously far from ideal being a solid tumour with a very different tumour microenvironment to that of a blood-derived cancer. Therefore one should be careful in translating the findings obtained here to leukaemia. Nonetheless, results showed that the combination of HAGE- and WT1-ImmunoBody^®^ was able to significantly delay tumour growth and prolong mouse survival when compared to mice in the control group. Indeed, mice that did not receive the vaccines exhibited faster growing tumours than those that received the vaccines and had to be sacrificed earlier due to the endpoint tumour size being reached. Interestingly, while the vast majority of mice in the prophylactic group developed a slow growing tumour, a distinct group of four mice exhibited faster tumour growth and these had to be culled earlier than the remaining six mice. Having seen such pattern of tumour development detected by bioluminescent imaging, it would be important, in the future, to continue administering the vaccines to the animals showing such a trend before the tumour size becomes too big. The mice in the therapeutic group exhibited a reduction in tumour size while the immunisation regime was ongoing, but as soon as the last injection of the vaccine was performed, the tumour started to grow, highlighting the fine balance between the efficacy of the vaccine and the increasing immunosuppression of the microenvironment as described by Schreiber’s group (39).

Comparing the outcomes of the present study with our previous studies using either HAGE- or WT1-ImmunoBody^®^ vaccination programme as a monotherapy (data not shown), the combined HAGE/WT1 ImmunoBody^®^ vaccines used herein were found to further improve the delay in the tumour growth in pre-immunised mice, but also and importantly, to delay tumour growth in a significant proportion of mice even when the vaccines were used after tumour implantation. Significance was not achieved when either vaccine was used on their own. These observations highlight the importance of incorporating epitopes from both HAGE and WT1 epitopes in future immunotherapy strategies. Moreover, this novel combination was found to induce notable HAGE- and WT1-specific T cells in the tumour bearing mice which were culled later in the experiment. This was in comparison to the mice that were euthanised early in the study in order to assess the IFN-γ ELISpot assay on splenocytes derived from the immunised mice so examination could occur before too much immunosuppression could interfere with the results.

Immunophenotypic analysis on TILs and splenocytes isolated from mice from different groups using flow cytometry demonstrated a clear reverse in the CD4^+^/CD8^+^ ratio of TILs in comparison with those derived from the spleens. Whereas the majority of CD3^+^ T cells in the spleens were CD4^+^ (80%), more than 75% of them were CD8^+^ T cells in the tumours. The high percent of recruited CD8^+^ cells were found in both vaccinated and non-vaccinated mice, thereby indicating that B16 tumour cells were being recognised as non-self probably due to the presence of HAGE and the luciferase reporter gene; the latter has been reported to induce immunogenicity in mice tumours (40, 41). However, the frequency of CD8^+^ TILs isolated from mice that were vaccinated with combined vaccines in the prophylactic and the therapeutic settings was significantly higher than those derived from the control group, indicating that these cells were the principal T cell type involved in controlling tumour growth.

We also noted that the CD4^+^ and CD8^+^ TILs that trafficked through the _h_B16/HAGE^+^/Luc^+^ tumour model exhibited a remarkable upregulation of PD-1 that was independent of the immunisations. PD-1 expression is promptly induced on T cell after TCR activation (42), and this expression is temporary, and declines when immunogen is cleared. PD-1 is, however, maintained and becomes persistently expressed on antigen-specific T cells in cancer or chronic disease (43) leading to functional impairment of CD8^+^ T-cells (44). As B16 expresses PD-L1 (PD-1 ligand), it is possible that the PD-1/PD-L1 pathway was activated and that this could be attributed to the modest efficacy noticed in the therapeutic setting. However, the prophylactic setting demonstrated significant benefits in terms of prolonged mouse survival and resistance to tumour growth, despite the upregulation of PD-1 on TILs. This suggests the occurrence of other mechanisms that could enable the combined vaccines in the prophylactic treatment to overcome PD-1/PD-L1 pathway activity. Because of the modest response seen in the therapeutic setting, we would recommend the incorporation of anti-PD-1/anti-PD-L1 in combination with novel vaccines to study whether a further enhancement of the anti-tumour effect that could be obtained.

## Conclusions

The present study revealed that the combined HAGE and WT1 vaccine was more effective than either vaccine alone, demonstrating the superiority of combination HAGE- and WT1- ImmunoBody^®^ vaccines. CTLs generated using this combination were also shown to specifically recognise and kill relevant targets.

The WT1 sequence used in this study was completely human and differs from the murine WT1 by a single amino-acid. Although this had no consequence when used in double transgenic mice, the same vaccine failed to generate any detectable immune responses in syngeneic C57BL/6 mice (Joshua Pearson, personal communication).

Although the observed delayed growth of the implanted tumour cells was only significant in the prophylactic study, these results also showed that the mice would have benefitted from a more sustained vaccination strategy. Moreover, the fact that B16 cells are PD-L1 positive and that CD3^+^ T cells were also positive for PD-1, means that one should consider continued vaccinations post-tumour implantation, as well as consider the inclusion of anti-PD-1 antibody therapy.

Overall, the data presented herein support the development of vaccine strategies based on a combination of both HAGE- and WT1-ImmunoBody^®^ vaccines which could be further improved with the use of immune checkpoint inhibitors.

## Data Availability Statement

The original contributions presented in the study are included in the article/**Supplementary Material**. Further inquiries can be directed to the corresponding author.

## Ethics Statement

Pre-clinical studies were approved by the Home Office under the Animals (Scientific Procedures) Act under two Project Licences (PPL): 1. 40/3563 valid until the 5th December 2016 2. PB26CF602, granted on the 28th of November 2016 and valid until the 28th of November 2021.

## Author Contributions

RA and SM contributed to the conception and design of the study. RA preformed the experiments. RA, SM, DN and JV contributed to the development of the methodology. SM and SR helped by labelling targets with chromium, RA and SM analysed the data and wrote the paper. VB, RM and LD provide the vaccines. AP and B-aG revised and corrected the manuscript. All authors contributed to the article and approved the submitted version.

## Conflict of Interest

LD is a director and shareholder of Scancell Ltd and is a named inventor on the ImmunoBody patents. VB and RM were employed by Scancell Ltd.

The remaining authors declare that the research was conducted in the absence of any commercial or financial relationships that could be construed as a potential conflict of interest.

## Supplementary Material

Supplementary Figure 1 HAGE and WT1 expression in CML stem cells and normal stem cells. CML single cell (SC) gene expression data was downloaded from GEO database (Acc. No. GSE76312, Giustacchini et al 2017), All data from single cell CML cell line were removed from the matrix prior to the analysis. Single cell gene expression data was available for 1964 patient derived CML cells and 232 Normal Hematopoietic cells (HSC). Gene expression values (RPKM) of CD34, CD38, HAGE and WT1 genes were extracted from the data matrix and matched up with the patient identities and clinical information. Values greater than 1RPKM were considered as a positive expression. Both HAGE and WT1 expression on single cells was shown to vary within patients with some cells express very high level of HAGE while others do not. No expression was detected in HSC cells for both WT1 and HAGE expression highlightinh their tumour cell specific expression.

Supplementary Figure 2 Sequential, real-time in vivo analysis of tumour burden in live animals assessed by Perkin Elmer IVIS Lumina III system. The figure reflects intra-tumoral luciferin bioluminescence signals in anesthetised HHDII/DR1 mice bearing _h_B16/HAGE^+^/Luc^+^ tumour. Images from different groups point out a decline in tumour size and prolonged mice survival in vaccinated group in comparison with control. Colours overlying mice represent the rate of photons emission of the luciferin per second, wherein, red refers to the highest photons density and violet corresponding to the least detectable emission.

## Abbreviations

Allo-SCT, allogeneic-stem cell transplantation; HAGE, helicase antigen; DDX43, DEAD-Box Helicase 43; WT1, Wilms tumour; CML, chronic myeloid leukaemia; TKIs, tyrosine kinase inhibitors; ER, endoplasmic reticulum; FCS, Foetal calf serum; β2m, beta2 microglobulin; TAPs, transporter-associated proteins; DMSO, Dimethyl sulfoxide; BFA, Brefeldin A; RT-qPCR, Real-time quantitative polymerase chain reaction; CT, cycle threshold; MFI, mean fluorescence intensity; FIR, fluorescence intensity ratio; PI, propidium iodide; FITC, fluorescein isothiocyanate; ^51^Cr, ^51^Chromium; ELISpot, Enzyme-linked immunospot; IVS, *in vitro* peptide re-stimulation; PD-1, programmed death-1; shRNA, Short hairpin RNA.
