# Supplementary figures and images for: A Novel HAGE/WT1-ImmunoBody® Vaccine Combination Enhances Anti-Tumour Responses When Compared to Either Vaccine Alone
Source: Front Oncol. 2021 Jun 28;11:636977. doi: 10.3389/fonc.2021.636977 (PMC8273701; doi:10.3389/fonc.2021.636977)

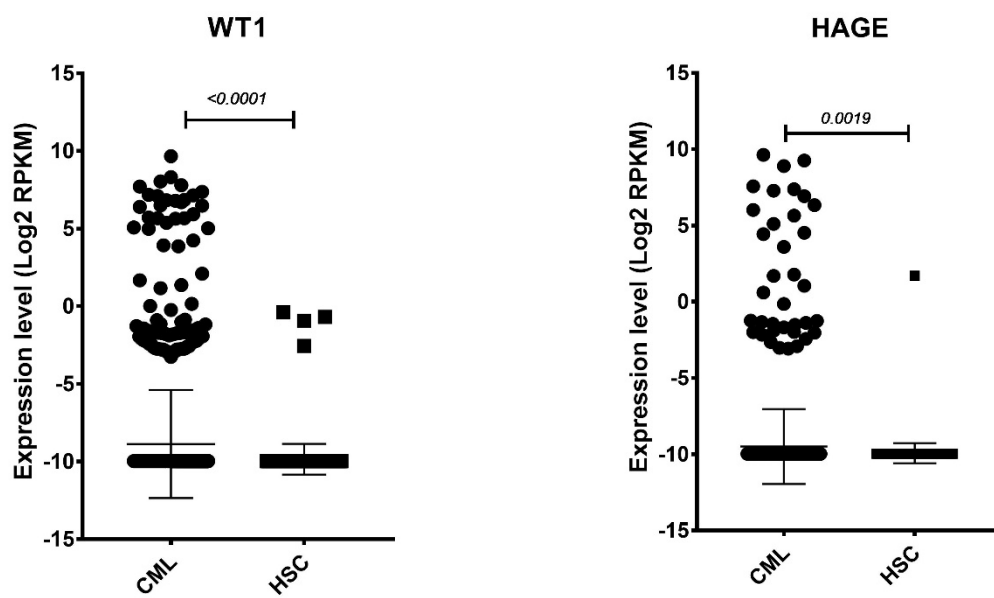

Suppl. Figure 1

Supplement: Supplementary Figure 1 — HAGE and WT1 expression in CML stem cells and normal stem cells. CML single cell (SC) gene expression data was downloaded from GEO database (Acc. No. GSE76312, Giustacchini et al 2017), All data from single cell CML cell line were removed from the matrix prior to the analysis. Single cell gene expression data was available for 1964 patient derived CML cells and 232 Normal Hematopoietic cells (HSC). Gene expression values (RPKM) of CD34, CD38, HAGE and WT1 genes were extracted from the data matrix and matched up with the patient identities and clinical information. Values greater than 1RPKM were considered as a positive expression. Both HAGE and WT1 expression on single cells was shown to vary within patients with some cells express very high level of HAGE while others do not. No expression was detected in HSC cells for both WT1 and HAGE expression highlightinh their tumour cell specific expression. [file Image_1.pdf]

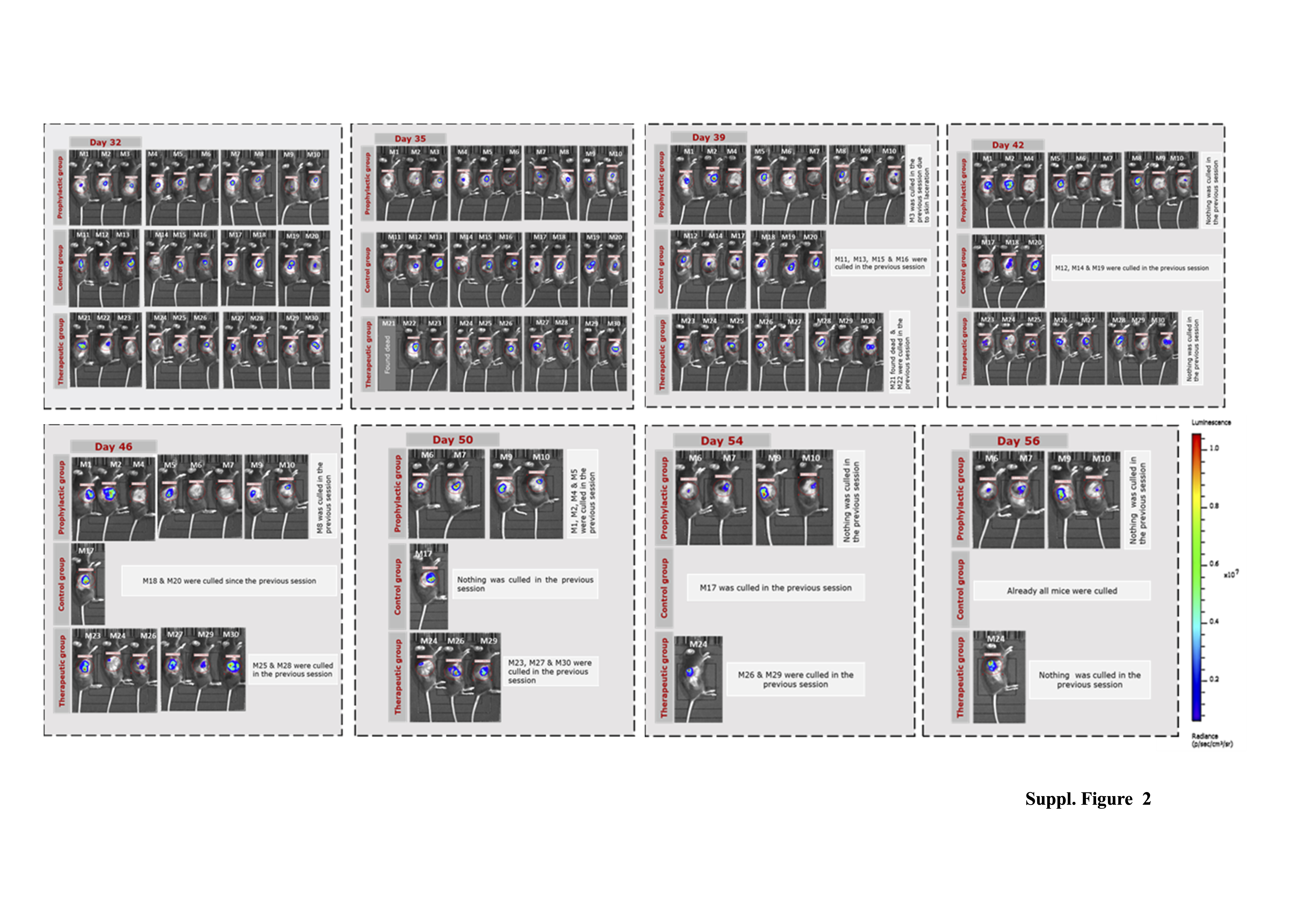

Supplement: Supplementary Figure 2 — Sequential, real-time in vivo analysis of tumour burden in live animals assessed by Perkin Elmer IVIS Lumina III system. The figure reflects intra-tumoral luciferin bioluminescence signals in anesthetised HHDII/DR1 mice bearing hB16/HAGE+/Luc+ tumour. Images from different groups point out a decline in tumour size and prolonged mice survival in vaccinated group in comparison with control. Colours overlying mice represent the rate of photons emission of the luciferin per second, wherein, red refers to the highest photons density and violet corresponding to the least detectable emission. [file Image_2.jpg]

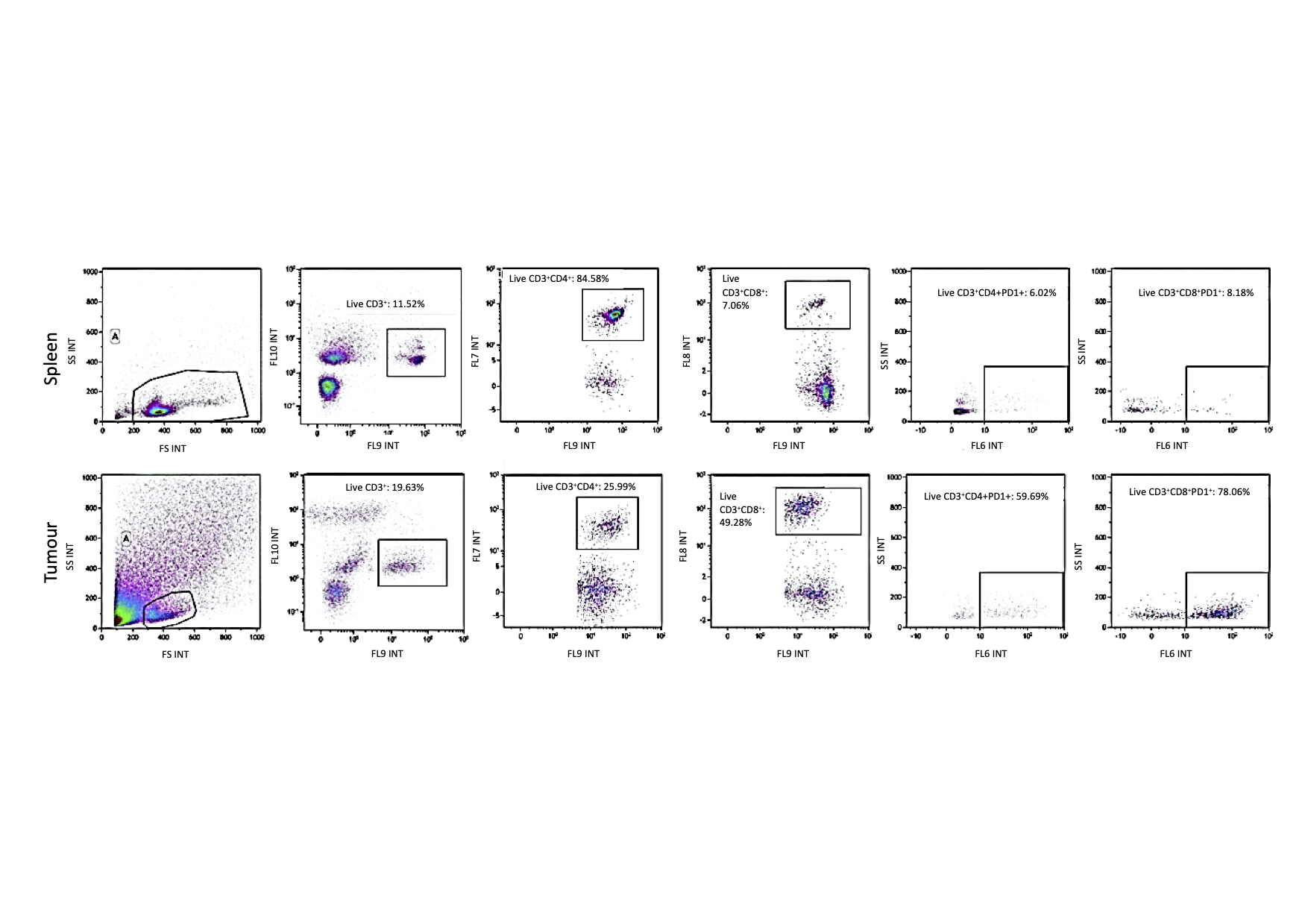

Supplement: Supplementary file 3 [file Image_3.jpeg]
